# Wear Morphology on the Surfaces of CoCrMo Unicompartmental Knee Joint Endoprostheses as Elements of Metal–Metal Friction Nodes

**DOI:** 10.3390/ma13122689

**Published:** 2020-06-12

**Authors:** Arkadiusz Szarek, Grzegorz Stradomski, Justyna Łukomska-Szarek, Dariusz Rydz, Wojciech Wolański, Kamil Joszko

**Affiliations:** 1 Faculty of Mechanical Engineering and Computer Science, Department of Technology and Automation, Czestochowa University of Technology, 21 Armii Krajowej Av., 42-201 Czestochowa, Poland; arek@iop.pcz.pl; 2 Faculty of Production Engineering and Materials Technology, Czestochowa University of Technology, 19 Armii Krajowej Av., 42-201 Czestochowa, Poland; gstradomski@wip.pcz.pl (G.S.); dariusz.rydz@pcz.pl (D.R.); 3 Faculty of Management, Czestochowa University of Technology, 19 B Armii Krajowej Av., 42-201 Czestochowa, Poland; j.lukomska-szarek@pcz.pl; 4 Faculty of Biomedical Engineering, Department of Biomechatronics, Silesian University of Technology, 40 Roosevelta Av., 41-800 Zabrze, Poland; wojciech.wolanski@polsl.pl

**Keywords:** endoprostheses CoCrMo, surface wear, microstructure

## Abstract

The article assesses the strength and structural parameters of load-bearing layers of metal biobearings made of CoCrMo alloy. The research material consisted of unicompartmental knee joint endoprostheses used in the human body, removed due to excessive wear. No patient participated in the examinations. The endoprostheses used as research material underwent the liquidation procedures in the hospital, which has all necessary permissions and certifications to perform endoprosthetic procedures. Endoprostheses selected for the examinations had been used for 6 to 12 years at similar load conditions as declared by the patients, i.e., body weight of F = 835 N, declared activity expressed as the number of load cycles up to 100 thousand/year, and no artificial joint infections. To assess the homogeneity of the research material, the analysis of chemical composition using a Joel scanning electron microscope with EDS (Energy-dispersive X-ray spectroscopy) was made to exclude endoprostheses with various alloying additives. Microscopic examinations were performed using the Phenom XL microscope, while the wear surface was examined using a Keyence VHX-900F microscope. Several experimental tests were also carried out on load-bearing surfaces to assess changes in strength parameters of the base material after a known life cycle and load conditions. Material hardness using the Vickers method, yield point, critical value of stress intensity coefficient, and the coefficient of friction µ were evaluated. The examinations allowed for the systematization of wear in the knee and femoral components of unicompartmental hip endoprostheses. The statistical evaluation of the number and costs of hip joint replacement surgeries in Poland was also made.

## 1. Introduction

Clinical and biomechanical research has shown [1,2,3,4] that in recent years, mechanical damage to implants, i.e., cracks or fractures, are no longer as a serious problem as the wear of friction surfaces of both knee and hip endoprostheses. The problem exists in various configurations of friction pairs, i.e., metal–UHMWP (Ultra-high-molecular-weight polyethylene), ceramics–UHMWP, metal–metal, and ceramics–ceramics, although the extent of wear depends directly on the type of the materials in contact. Therefore, it can be concluded that currently, the biggest problem is the wear of artificial joint components [1,2,3,4]. The percentage of reimplantations of knee joint endoprostheses has remained for years at an unsatisfactory level of 5% [5], and since the number of implanted knee joint endoprostheses in Poland and worldwide is rapidly growing [6,7,8,9,10,11,12,13,14] research is being carried out to extend the life of endoprosthesis parts in the human body [15,16,17,18]. The tribological parameters in replacement joints, despite the use of modern biomaterials, differ significantly from the material of the natural human joint. The use of polymer inserts ensures the proper biomechanics of the artificial joint. However, this is true only within a short period after the implantation. Therefore, the high degradation of the material, and wear products migrating to the body, force companies to look for alternative material solutions for artificial biobearings. Until recently, it seemed that the optimal solution was to use metal–metal friction pairs, but the clinical practice has shown that the joint surfaces are also damaged with use in such solutions [19,20,21,22,23,24,25]. Therefore, an attempt was made to determine the mechanism of destruction of bearing surfaces in an artificial joint. Based on the elements removed from the human body, a representative group of endoprostheses with the same chemical composition used in similar conditions and with a known time of use was selected and an attempt was made to determine and systematize the pattern of destruction of the surface of an artificial biobearing.

It was determined based on the experiments that a group of examined components is characterized by similar chemical composition. Therefore, the fact that the endoprostheses worked under comparable conditions allows for the description and systematization of the wear processes depending on the period of prosthesis use. Endoprostheses obtained as a result of reimplantation are made of one of the most popular alloys based on CoCrMo used for the elements of artificial biobearings, characterized by much higher strength parameters than other materials used for prostheses, e.g., Ti6Al4V or UHMWPE.

One problem resulting from the introduction of metal elements into the human body is the fact that metals are characterized by a significant immunogenic potential and are among the primary causes of contact allergy. The study [26] documented inflammatory and allergic responses induced by metals in patients following the implantation of various types of implants. It was shown that nickel, chromium, and cobalt ions can be released from the damaged prosthesis, especially if the chromium oxide coating is worn [26]. Complications following the total hip arthroplasty (THA) and knee joint arthroplasty (TKA) are reported in ca. 5% of patients [27], which, with 773,813 endoprostheses implanted in Poland only in the last five years, makes a considerable number of 38,691 [8,9,10,11,12,13,14].

Components of endoprostheses, which represent the load-bearing systems that transfer loads resulting from the biomechanics of individual joints, have for many years been manufactured using technologies allowing for obtaining significantly improved material strength parameters. Consequently, cracks and damages are relatively rare in endoprostheses, although they were common in the initial period of the mass use of components for joint replacement. A much greater prevalence of damage to artificial joints results from its tribological wear. This type of damage occurs in all types of joints and concerns the surfaces in contact which constitute a biobearing expected to show the kinematic parameters and load-bearing capacity typical of the joint.

The wear of prosthesis components occurs both chemically and mechanically. Chemical wear is affected by the biomaterial used and ion concentration observed in the human body. Mechanical wear depends in particular on the patient’s body weight, the intensity of use, range of motion in the artificial joint, and accuracy of implantation of individual components [28].

**Figure 1:**
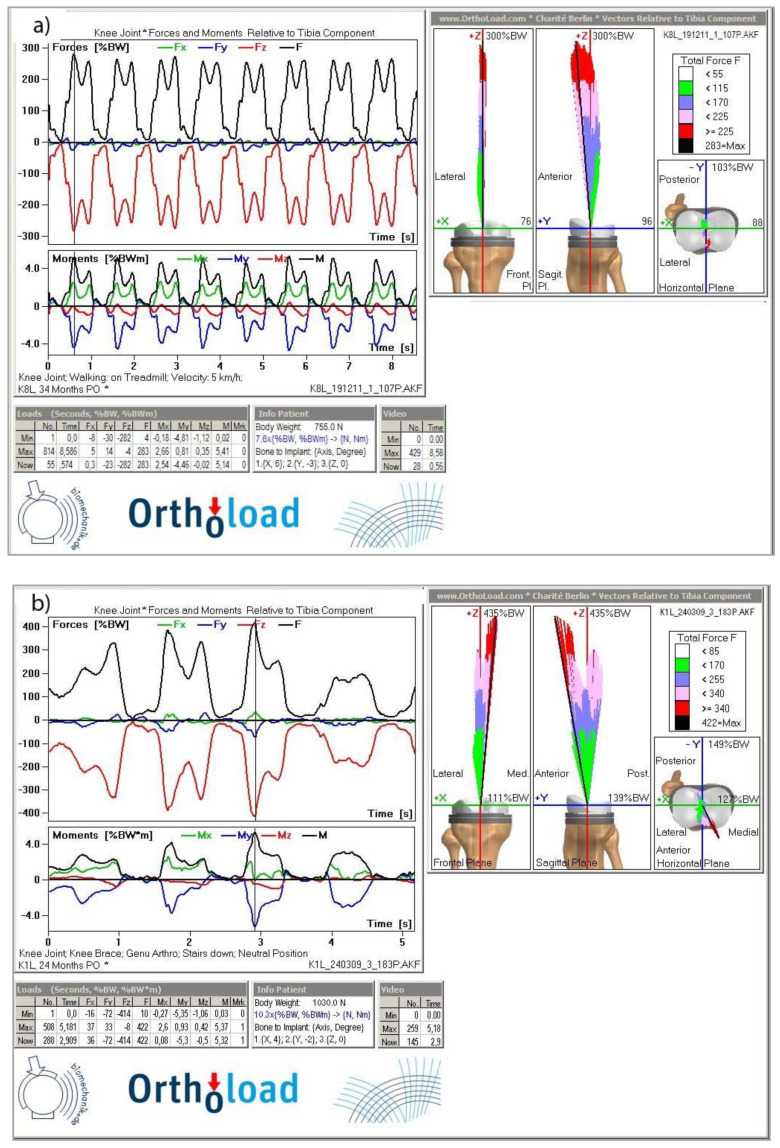
Effect of forces acting on the knee joint: (**a**) walking at the speed of 5 km/h (**b**) going downstairs [29,30].

It can be observed that during walking at a speed of 5 km/h (see Figure 1a), the net force reaches on average 283% of body weight. Significantly higher overload occurs in the knee joint when the patient is going downstairs, see Figure 1b. A forward displacement of the center of gravity and an impact load on a flat surface from a height equal to that of the stairstep height generates a knee overload of 410% BW, which is 153% of the load observed during gait, see Figure 1.

From a biomechanical standpoint, the greatest loads are transferred through the knee joint during human motor activity. During gait, cyclic loads are repeated at specific time intervals. The maximum load on the knee joint occurs when the leg touches the ground and decreases with the change of body position during the transfer phase [31,32,33]. To optimize the process of loading the prosthesis, tests are carried out over the full life cycle of the prosthesis, from the moment of its implantation through the correct process of adjusting the prosthesis during surgery [34,35] to biomechanical tests aimed to evaluate the load in prostheses during basic activities [36,37,38].

## 2. Statistical Evaluation of the Number of Surgeries and Costs of Joint Replacement in Poland

Cook et al. [6] pointed to the rapidly growing number of THA and total knee arthroplasty (TKA) surgeries worldwide, increased costs, and worse clinical outcomes of revision surgeries. As predicted by Singh, Yu, Chen and Cleveland [7], the number of joint replacement surgeries will be progressive between 2020 and 2040, although the most dynamic growth is forecast primarily for TKA. The number of THAs and TKAs per year is predicted to have increased in [7]:

- 2020: THA—by 34% (to 498,000 replacements); TKA by 56% (to 1,065,000 replacements);
- 2025: THA—by 75% (to 652,000 replacements); TKA by 110% (to 1,272,000 replacements);
- 2030: THA—by 129% (to 850,000 replacements); TKA by 182% (to 1,921,000 replacements);
- 2040: THA—by 284% (to 1,429,000 replacements); TKA by 401% (to 3,416,000 replacements).

**Figure 2:**
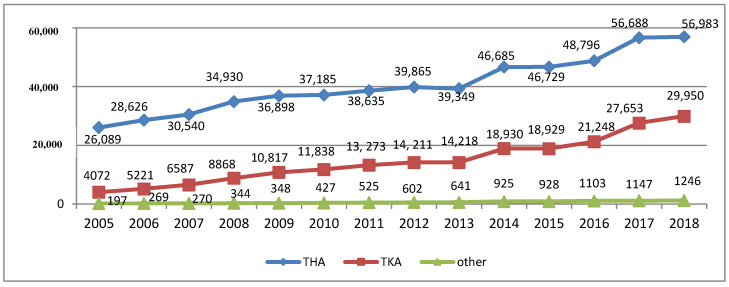
Number of individual types of joint replacement surgeries performed in Poland in 2005–2018 (author’s own study based on [8,9,10,11,12,13,14]).

Recent years have also seen a rapid increase in the number of joint replacement surgeries in Poland. In 2018, a total of 88,179 of such surgeries were recorded in Poland (30,378 in 2005, which was an increase of 290%), of which 56,983 concerned the hip joint, 29,950 the knee joint, 767 the shoulder joint, and 230 the elbow joint [8,9,10,11,12,13,14]. Detailed data on the number of THAs and TKAs are shown in Figure 2. 

**Figure 3:**
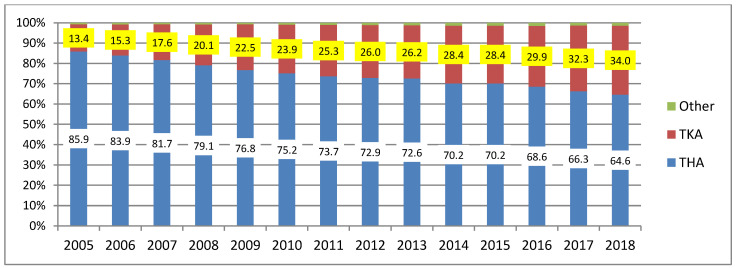
Percentage of individual types of joint replacement surgeries performed in Poland in 2005–2018 (author’s own study based on [8,9,10,11,12,13,14]).

The highest number was observed for hip joint replacement surgeries performed in 2005–2018, although with a steadily decreasing trend, amounting for 85.9% of all joint replacement surgeries in 2005, and 64.6% in 2018, which translated into a decrease of 21.3%. However, throughout the study, the escalation of knee joint replacement surgeries was observed as their percentage increased from 13.4% in 2005 to 34% in 2018, as shown in Figure 3.

**Figure 4:**
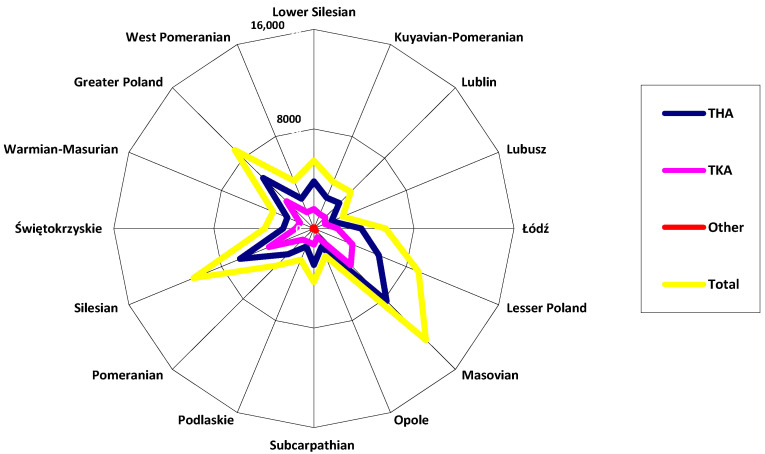
Number of joint replacement surgeries performed in 2018 per voivodeship in Poland (author’s own study based on [14]).

It can be observed based on the data illustrated in Figure 4 concerning the number of joint replacement surgeries performed in 2018 per voivodeship in Poland that hip and knee joint surgeries were most prevalent. The highest number of surgical procedures was recorded in the Masovian Voivodeship (a total of 12,659 joint replacement surgeries, of which the knee joint surgeries accounted for 4178), the Silesian Voivodeship (10,399 joint replacement surgeries, of which there were 3887 of knee replacement surgeries), the Lesser Poland Voivodeship (9057 joint replacement surgeries, of which the number of knee joint surgeries was 3343), and the Greater Poland Voivodeship (8922 surgeries, with 3084 knee joint surgeries).

**Figure 5:**
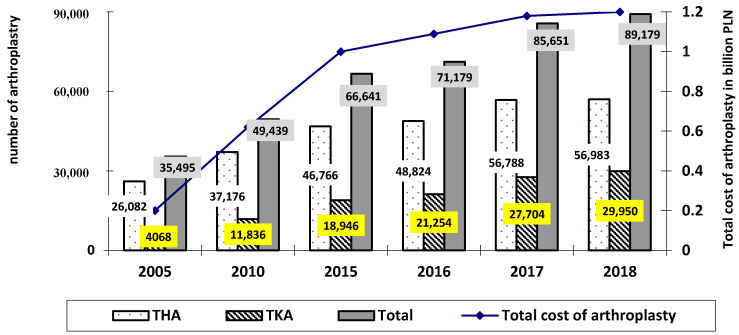
The number and costs of joint replacement surgeries performed in 2005–2018 (author’s own study based on [8,9,10,11,12,13,14]).

The costs of joint replacement surgeries in Poland were also growing, from 0.2 billion PLN in 2005 to over 1.2 billion PLN in 2018 [8,9,10,11,12,13,14]. Figure 5 presents the number and costs of joint replacement surgeries performed in 2005–2018. In Poland, the costs of individual types of joint replacement surgeries in 2005–2018 were on average (author’s own study based on [8,9,10,11,12,13,14,39,40]; using the mean exchange rate as of 30 December 2005 of 1 EUR = 3.86 PLN and 1 USD = 3.26 PLN; using the mean exchange rate as of 30 December 2018 of 1 EUR = 4.3 PLN and 1 USD = 3.76 PLN):

(1) hip joint:
  - cement alloplasty: in 2005: 9933–11,100 PLN (which translated into 2573–2876 EUR and 3047–3405 USD), in 2018: 10,000–11,700 PLN (which translated into 2325–2720 EUR and 2659–3112 USD);
  - cementless arthroplasty: in 2005: 12,220–13,000 PLN (which translated into 3161–3368 EUR and 3742–3988 USD), in 2018: 13,000–23,000 PLN (which translated into 3023–5349 EUR and 3457–6117 USD);
  - revision arthroplasty: in 2005: 20,835–24,095 PLN (which translated into 5398–6242 EUR and 6391–7391 USD), in 2018: 19,000–20,000 PLN (which translated into 4419–9070 EUR and 5053–5319 USD).
(2) knee joint:
  - cement arthroplasty: in 2005 13,590–17,456 PLN (which translated into 3521–4522 EUR and 4169–5355 USD); in 2018: 15,750–20,000 PLN (which translated into 3662–4651 EUR and 4189–5319 USD);
  - revision arthroplasty: in 2005: 21,715–23,568 PLN (which translated into 5626–6106 EUR and 6661–7229 USD); in 2018: 12,500–20,000 PLN (which translated into 2907–4651 EUR and 3324–5319 USD).

In conclusion, the number and costs of joint replacement surgeries performed in 2005–2018 in Poland were growing dynamically, especially for the knee joint. The worldwide escalation of knee joint replacement surgeries is also expected to take place between 2020 and 2040 (from 56% to 401%) [7]. The research on strength parameters of materials used in the production of knee joint prostheses may lead to a significant extension of prosthesis life and a decrease in the number of replacement surgeries.

## 3. Materials and Methods

The research material consisted of two new samples (unused), three samples used in the human body for about 6 years, and one sample used in the body for 12 years.

**Figure 6:**
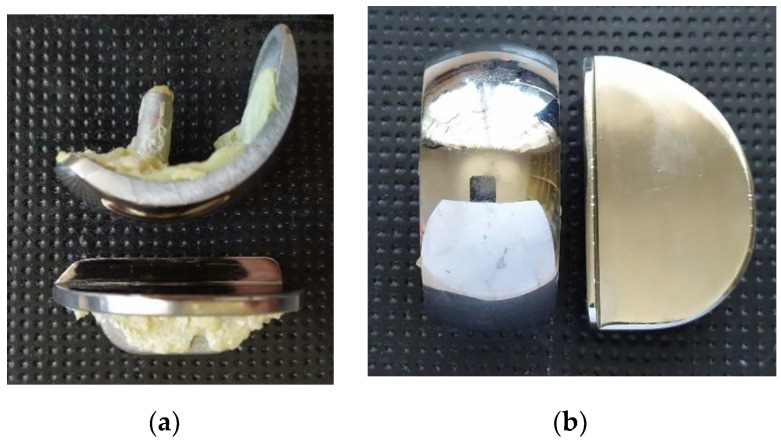
Research material: unicompartmental knee joint endoprosthesis, (**a**) components of endoprostheses, (**b**) the surfaces of a metal–metal contact.

The experimental examinations conducted using a Joel + EDS scanning microscope were used to perform a chemical analysis of the elements present in prostheses removed from artificial joints implanted in the human body. The quantitative analysis of the elements from a selected area and evaluation of the distribution of elements in microareas revealed that cobalt-based implants are characterized by the following chemical composition (see Table 1):

The surfaces of a metal–metal contact in half of knee joint endoprosthesis usually include a flat tibial insert and a femoral component with a complex contact shape. The unicompartmental knee joint endoprosthesis used in the study is shown in Figure 6a,b.

Endoprostheses are mainly characterized by two contact surfaces. Macrogeometry of contact reflects the anatomical shape of the joint surface, whereas microgeometry should be characterized by perfectly smooth surface topography. Actual surfaces of joint prostheses are not perfectly smooth and are characterized by an uneven but measurable deviation from the ideal profile. These deviations have a significant effect on the mechanics of contact between endoprostheses and are related to the shape and geometric structure of the surface.

Knee movement is a combination of rolling and sliding motion. A rolling motion occurs in the first phase of the movement. With further flexion, the femoral condyles roll and at the same time slide over the joint surface of the tibial bone condyles. In the final phase of flexion, the femoral condyles slide without the rolling movement. The lateral condyle of the femur rolls slightly further than the medial one [41].

A detailed analysis of the phenomena observed in joints is very difficult and complicated because the conditions such as pressure, viscosity, and the condition of the joint surface may change substantially. The studies by previous authors, including [42,43], have shown that four basic types of friction are observed in human joints: fluid friction, bioelastohydrodynamic friction, mixed friction, and boundary friction. The synovial fluid between joint surfaces minimizes the wear of joint surfaces and reduces the value of the coefficient of friction. Assuming that two stiff bone surfaces are in contact in the joint and that the fluid viscosity is constant, a value obtained for the lubricating film gap was g = 0.01–0.02 µm [42,44]. Given the actual surface roughness in the cartilage Ra of 0.02–0.2 µm, and based on the elastohydrodynamic lubrication theory, the thickness of the film between the surfaces in contact is g = 10–20 µm [42,45]. With this complicated structure, the coefficient of friction typical of natural synovial joints for healthy and unaffected human joints is very low. For the knee joint, its value amounts to µ = 0.005–0.02, while for the hip joint, this coefficient is µ = 0.001–0.03 [42,46].

In clinical practice, the metal–metal friction pair is increasingly being used for artificial joint systems, hoping to extend the service life. However, the process of destruction and migration of wear products can have numerous dramatic consequences. The wear products in metal–metal joints are transported to soft tissues, thus leading to inflammation and metallosis. In the clinical practice, this is frequently diagnosed as pseudotumors leading to the recommendation for prosthesis reimplantation. The migration of the wear products can also cause dangerous complications. If particles of worn material accumulate inside the joint, extensive intraarticular infections may occur. However, if the products move to the liver, pancreas, or kidneys, their function may be damaged or the structures that oncologists diagnose as cancer may be formed. In extreme cases, if wear products enter the brain, this can even lead to the patient’s death. Taking into account the above, it is very important to determine the pattern of the process of destruction of metal elements implanted inside the joint.

## 4. Results and Discussion: Assessment of the Destruction of Surfaces of Unicompartmental Endoprostheses

In the first phase of this study, an attempt was made to evaluate the wear of contact surfaces using microscopic tests. The metrology of stereometric characteristics of the surface is already developed to such an extent that it is possible to predict the behavior of the surface of a component in contact with another one and the extent to which it will perform its intended functions during use [47,48]. Surface topography was measured based on microscopic examinations using a Keyence VHX-900F microscope (Keyence company, Osaka, Japan). The analysis and evaluation of wear of the contact surfaces in the artificial knee joint used for 6 years was performed in the identified area of wear in three samples. The test results were compared with the results of the sample used in the human body for 12 years. In macroscopic and microscopic examinations, the research material used for 6 years was characterized by similar destruction of the surface.

**Figure 7:**
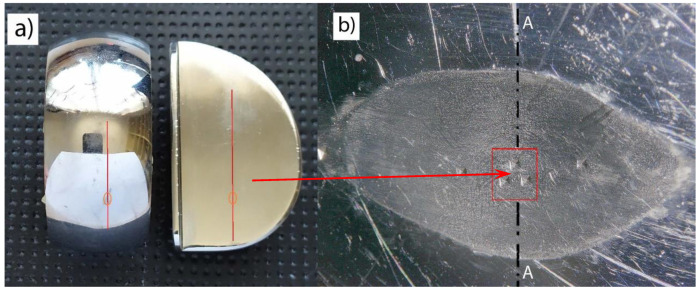
Area of wear: (**a**) hardness measurement line, (**b**) the area where the greatest increase in hardness was found, A-A: the cross-section of the sample used for microscopic examinations.

In the areas of wear, a wear line was drawn, on which the area of maximum wear (Figure 7a) was separated, and, in the area of maximum wear, HV_100_ hardness measurements were performed and samples were cut to identify the microstructure (section A-A), see Figure 7b.

The wear of endoprostheses components is of a chemical and mechanical nature, with the mechanical factors determining the intensity of wear being body weight, the intensity of use (daily number of steps), the roughness of implant surfaces, and range of motion in an artificial joint.

**Figure 8:**
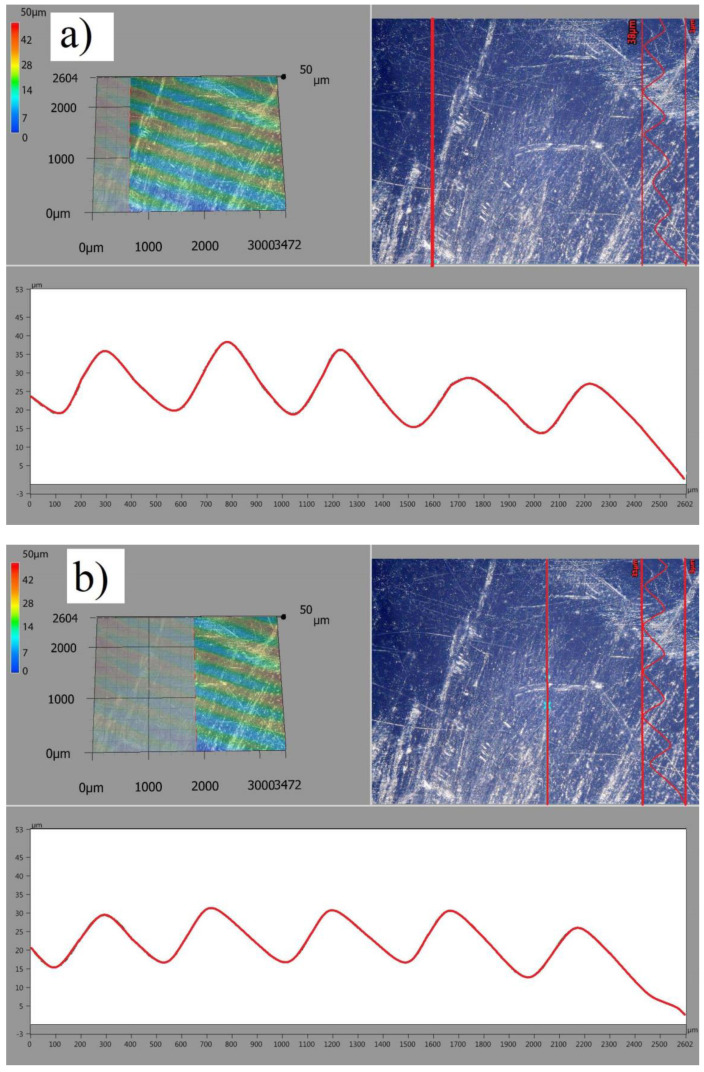

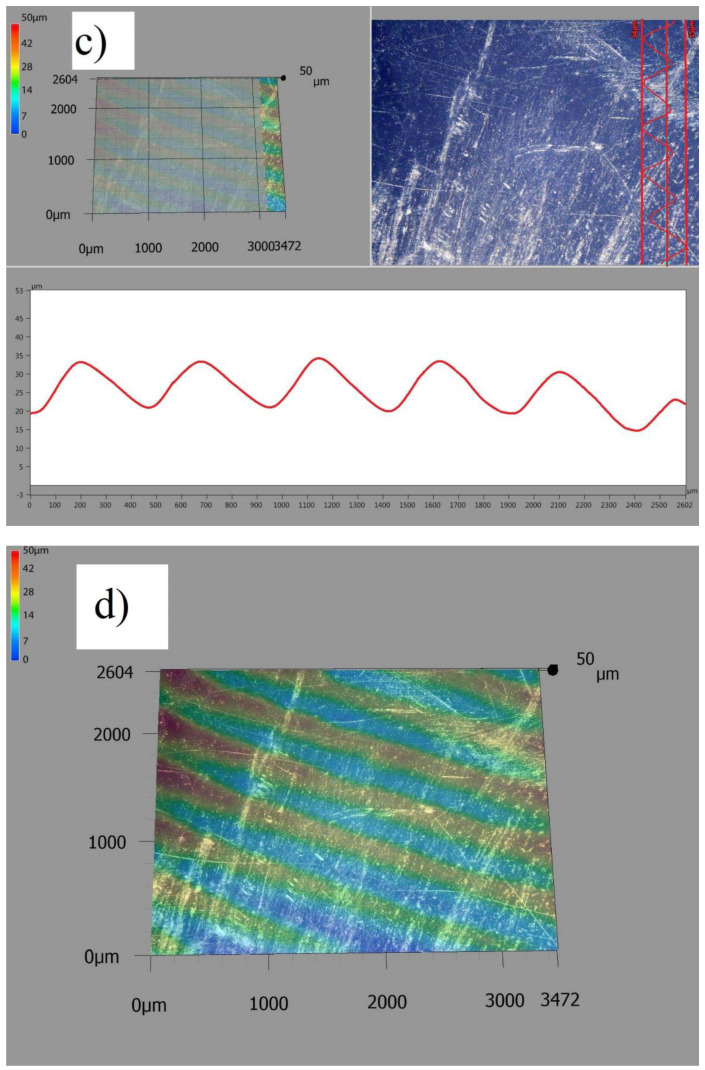
Scratch marks on tibial components after ca. 6 years of use, (**a**) surface topography at the beginning of wear, (**b**) surface topography in the middle of wear, (**c**) surface topography at the end of wear, (**d**) full surface topography.

In the initial period of use, the mechanics of contact surfaces during human motor activity is quite simple. The peaks of rough surfaces are elastically deformed and, after exceeding a certain value of pressure and friction resistance, plastic deformation of the peaks occurs, combined with their microabrasion. As a result of the cyclic fatigue load, the peaks will be remodeled to such an extent that the actual contact area will be large enough to transfer the external loads acting on the artificial joint. The process of rolling of the femoral component on the tibial part does not cause major changes during this period of use. However, the first surface scratches occur in the sliding area. The wear products are transported with the motion of the friction elements and accumulate in the final contact area. The surface image at 100× magnification with the surface profile is illustrated in Figure 8.

In the explorations period of use, the components being in contact wear against each other and, until optimal tribological conditions are achieved, the shape of the contact surfaces and mechanics of the surface layer change.

**Figure 9:**
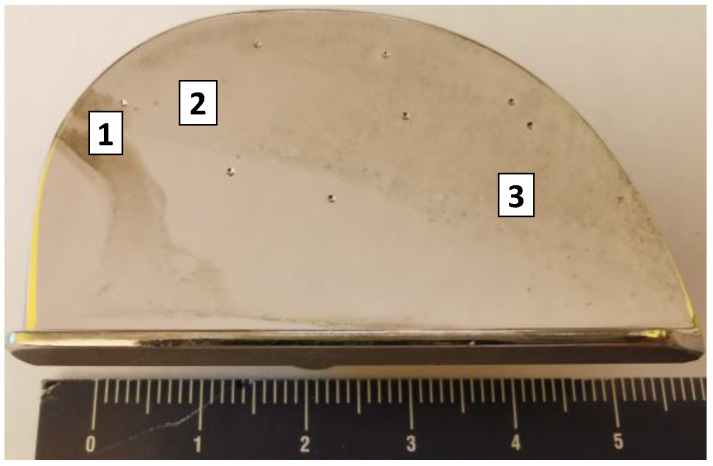
Indication of analyzed zone. (1- damage-free zone, 2- tribological wear zone, 3- zone surface with visible branched cracks).

**Figure 10:**
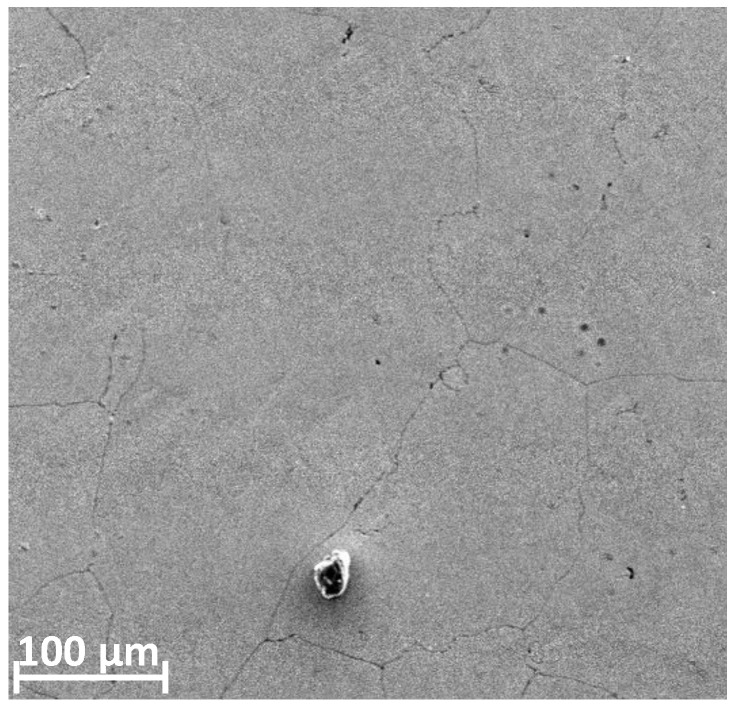
Topography of the endoprosthesis in the undamaged area with a single artefact.

**Figure 11:**
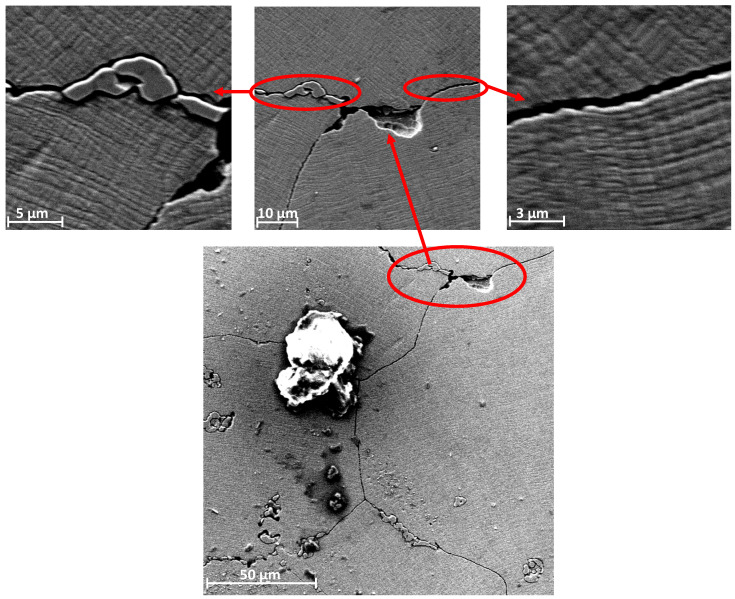
An exemplary view of the topography of the second zone surface with visible areas of destruction, artefacts, and cracks.

The microstructure and topography of the surface of the prostheses used were determined using a Phenom XL scanning microscope. Microscopic analysis revealed that there were three types of areas on the examined surface (Figure 9). The first one was the damage-free zone (Figure 10), characterized by no symptoms of frictional wear. No other damages such as cracks were found in this area and only single artefacts (transported from other places) were observed. However, there were no larger frictional wear zones with characteristic lines indicating the direction of frictional forces in this area. The second zone was the tribological wear zone, see Figure 11.

**Figure 12:**
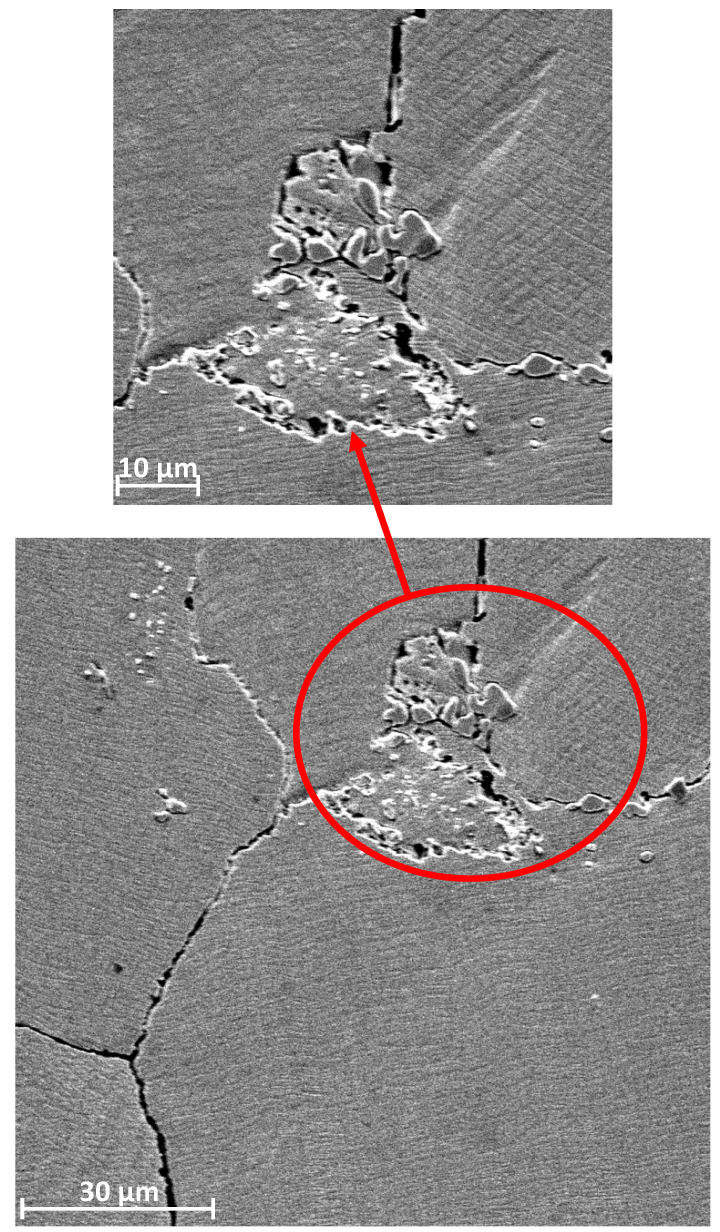
An exemplary view of the topography of the third zone surface with visible branched cracks and extended intergranular boundaries.

Noticeable lines are observed in this zone, located along the working direction. Similar to the first zone, this zone is also characterized by the presence of artefacts. Their number and size are much larger. Importantly, there is also typical fatigue chipping in this area. The last area is presented in Figure 11. This is the area of typical fatigue damage. This zone is characterized by the occurrence of slight pressing because of plastic deformation. Furthermore, substantially branched cracks are also observed. These cracks are usually of fatigue character. In addition to the cracks in grain parts, the increase in the width of boundaries between grains can be noted, which also indicates the occurrence of fatigue-type damage in this area. A frequent occurrence is the carbides pressed into the area of the grain boundaries, as illustrated in Figure 12. These carbides, as shown below, are observed in the entire volume of the material both at the grain boundaries and inside the grain. As a result of cyclic deformation, such hard-pressed material will not only chip and, as an artefact, but also be moved and pressed into another place, thus increasing local wear. It will also expose the intergranular boundary. The area remaining after such a carbide is a location of stress accumulation, which in turn has a destructive effect.

**Figure 13:**
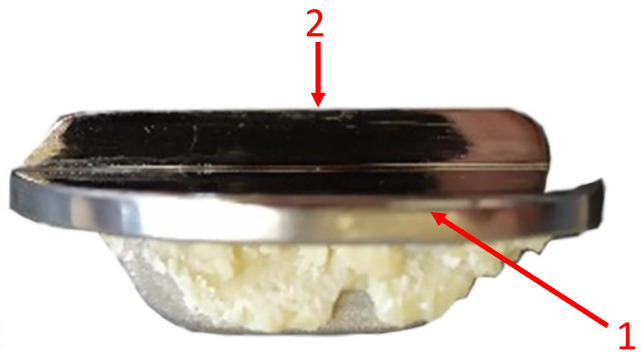
Samples for microstructure examinations.

**Figure 14:**
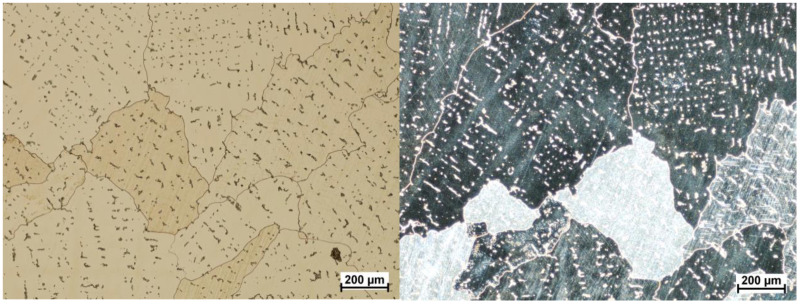
Microstructure area 1, light field on the **left**, dark field on the **right**.

**Figure 15:**
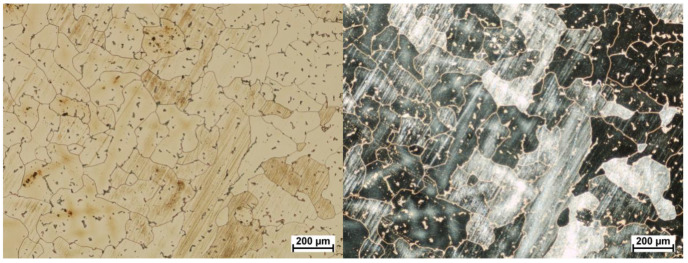
Microstructure area 2, light field on the **left**, dark field on the **right**.

The next part of the examinations involved cutting of the samples for microstructural analyses. the main purpose of this part of the research was to assess the correctness of the microstructure. This analysis was necessary because, testing the surface of wear only gave an answer about the changes in the surface layer. The observed changes of fatigue characteristics should also be reflected in the microstructure of the tested material. The cross-sections, etched with aqua regia (three portions of hydrochloric acid HCl and one portion of nitric acid HNO_3_), were sampled perpendicularly (Figure 13), which allowed for the assessment of the grain size and possible traces of cold plastic deformation during use. The examinations were conducted using the Nikon Eclipse Ma 200 microscope and the analysis was performed in a light and dark field. The microstructure of material is typical for and should be described as correct (β-Co phase + M_23_C_6_ carbides). In addition, should be mentioned that the material is characterized by equiaxed rather than columnar grains and no discontinuities were observed. An example of the microstructure of the material studied is shown in Figure 14 and Figure 15.

**Figure 16:**
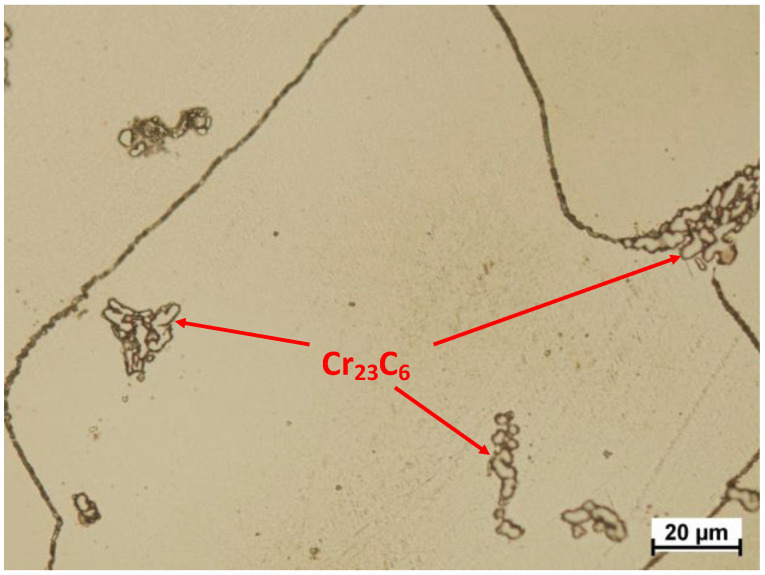
Distribution of carbides in the microstructure of the test material.

Microstructure analysis revealed a clear difference in grain size between the two zones. This demonstrates the differences in local strain rates during the manufacturing process. The zone 1 is the area just below the surface of frictional wear. It should be stressed that the observed large grains in zone 1 have both positive and negative effects. On the one hand, large grains increase their hardness, thus reducing tribological wear; on the other hand, micro deformations cause a higher stress build-up in the grain boundary area. Furthermore, the additional M_23_C_6_ type carbide secretions in this alloy including Cr_23_C_6_ carbides (Figure 16), are distributed both inside the grains and at their boundaries.

An interesting observation is that large grains in the first area, despite their local deformations (microdeformations) became a kind of barrier. No plastic deformation was observed in the subsurface zone, which indicates that due to large grains of ca. 600–800 μm, microdeformation causing local surface fatigue-type damage (carbide chipping and grain boundary widening) is located only in the surface layer.

In the analyzed period, the elastic deformation of the surface layer was observed during friction, which prevented structural changes and mechanical properties at the contact surface of the friction pair. Numerous scratches and damages resulting from tribological wear were diagnosed on the contact surface. The depth of the scratches was 20 µm, whereas the topography of the surface corresponds to the direction of the main movement of the endoprosthesis components. No transfer of the surface material or defects resulting from surface chipping were recorded. Similar scratches were found on the femoral component.

Chemical reactions between the wear products and in the area of tribological wear of a passive layer covering the implant and body fluids inside the body lead to plasticization of the implant load-bearing layer.

Human motor activity occurs at low speeds, usually not exceeding 3 km/h. This is also connected with very low speeds of displacement of the component of artificial joints in relation to each other. Friction in such a biobearing is characterized by very low speeds, which can be accompanied by self-excited vibrations that interfere with the friction. In addition, the resulting vibrations are transmitted to the component of the prosthesis, which is particularly unfavorable in the areas where the prosthesis is fixed to the bone. In addition, they move relative to each other, leading to microgrooves and disturbing the smoothness of movement. A constant and low relative movement speed of bodies subjected to friction cause the actual sliding speed to occur stepwise (stick-slip effect). A modification of this pattern of friction occurs each time after changing the parameters of the surface layer.

Cyclic fatigue loads cause the destruction to grain triple-points and brittle crushing of carbide fragments, which, combined with an increase in surface roughness, hardness, and frictional wear, intensifies the destruction of elements.

The wear starts to progress very rapidly from the moment when the intergranular destruction processes, crushing, and dispergation of the layer particles begin. Increasingly larger fragments are crushed, transported, and pressed into new areas of the load-bearing surface, forming a groove-like contact acting as a grinding wheel on the other component of the endoprosthesis. The wear of the components is then a combination of brittle cracking and layer dispergation and intensified frictional wear. Frictional wear products also migrate by sticking to the load-bearing surface of the prosthesis. This involves a radical change in strength parameters of the layer and a change in its surface topography.

**Figure 17:**
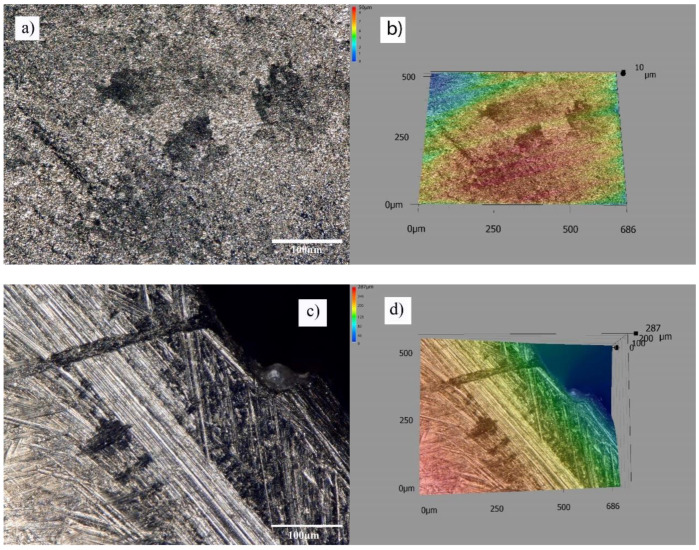
Image of wear of the artificial joint after 12 years of use, (**a**) material losses, (**b**) material losses 3d view, (**c**) frictional wear, (**d**) frictional wear3d view.

The surface image of the tibial component of the artificial knee joint operated for 12 years in the wear area at 500× magnification is shown in Figure 17a,b, whereas the image for the knee components is presented in Figure 17c,d.

The wear of the implant surface and a change in the surface roughness profile leads to changes in tribological parameters in the artificial joint.

The coefficient of friction determined in accordance with ASTM D1894 for the unused implant surfaces was µ = 0.25, whereas for surfaces with a modified layer after 12 years of use, it was µ = 0.47. The kinematics in such a destructively modified artificial joint is associated with significant frictional resistance, vibrations transmitted to the prosthesis elements, and a high release of wear products.

The wear is affected by the mechanical properties of the layer, which change with the intensification of the wear process. In tribological processes, hardness and yield point Re are of key importance. Basic mechanical parameters of endoprostheses were determined to assess the pattern of destruction of load-bearing surfaces (Table 2). Hardness tests using the Vickers HV_100_ method according to the PN-EN ISO 6507-1 standard were performed, and the yield point Re and the stress intensity coefficient K_IC_ were calculated. The tests were performed along the wear line, and the presented result was determined at the point of maximum wear Figure 7.

Hardness was measured along the contact line between the tibial and femoral components. In the case of new endoprostheses, the surface hardness for the tibial component was 360 HV_100_, whereas for the femoral component, it was 400 HV_100_. For endoprostheses used in the human body for ca. 6 years, the parameters did not change in an area that did not carry the loads resulting from the motor activity. However, numerous wear marks were observed on the contact surfaces of the two components. In the case of endoprostheses used for 12 years, permanent surface damage and destruction occurred in the load-bearing layer. The hardness measurement in the unloaded area remained unchanged, but there was a significant increase in hardness up to 430 HV_100_ in the wear area for the tibial component and 440 HV_100_ for the femoral component (Figure 7).

It can also be pointed out that the change in the mechanical parameters of the layer as a result of wear processes results in significantly different stresses on contact surfaces. The size and intensity of wear are also affected by the mechanical properties of individual materials, including resistance to brittle cracking.

A critical value of stress intensity coefficient K_IC_ was adopted as a measure of resistance to brittle cracking, which was calculated according to the formula (1) for individual stages of strengthening of the materials in contact:

(1) $$K_{\mathrm{IC}}={\left(\frac{E}{HV}\right)}^{0,5}\;\frac{F}{c^{1,5}}$$

where: *E*—Young’s modulus, *HV*—Vickers hardness, *F*—loading force, *c*—length of cracks propagating from the corners of the indentation.

It was found, based on the author’s research, that the values of stress intensity coefficient K_IC_ for the components used in the human body vary significantly depending on the period of use. As a result of cyclic fatigue load resulting from human locomotion, the contact surface of endoprostheses becomes more brittle. For the new tibial component, the stress intensity coefficient was K_IC_ = 106 [MPa m^1/2^], while after 6 years of operation, it reached K_IC_ = 100 [MPa m^1/2^]. The biggest change in the parameters could be observed in the period of use from 6th to 12th year, where the value of K_IC_ decreased to 77 [MPa m^1/2^]. For the femoral component, the stress intensity coefficient varied from K_IC_ = 126 [MPa m^1/2^] for the unused component to K_IC_ = 98 [MPa m^1/2^] after 12 years of use.

The strength parameters of the load-bearing layer change with the intensity of wear. However, this process is not uniform: during the initial period of use, i.e., up to about 6 years of use, changes in the macro and micro surface take place in terms of elastic material parameters. For the unused tibial component, Re = 120 [MPa], whereas for the femoral component, this value is Re = 133 [MPa] and, within comparable limits, it is maintained until the mechanical surface deformation caused by human motor activity. Abrasion in an artificial joint accelerates drastically with the intensification of changes in strength parameters of load-bearing surfaces. After ca. 8 years, the layer becomes brittle and less elastic, which makes the frictional wear and chipping of the material much greater and the wear process more intense. For knee components used in the body for 12 years, the yield point of the tibial component in use is Re = 143 [MPa], while for the femoral component, it is Re = 145 [MPa], which means nearly 10% to 20% increase in yield point and hardness of the contact layer due to chemical and mechanical wear. This has a significant effect on irreversible microscopic plastic deformations in all grains and at their boundaries, which causes them to be pulled out of the material and transported to other areas where agglomerates pressed into the native material of the prosthesis are formed. As a result of use, the surface layer becomes more brittle, it is fragmented, whereas the chipped particles are transported to other areas of the layer and mixed by dispergation.

## 5. Conclusions

The most common factor causing the necessity of reimplantation (replacement) of the artificial joint is wear of the load-bearing surfaces of the endoprosthesis components.

The main wear factor is the intensity of use of the artificial joint and the patient’s body weight. After about 6 years, the material parameters of the surface layer change, which leads to an increase in surface hardness and an increase in yield point. Our research found that the mean hardness HV_100_ on the surface of the new tibial component is 360 HV_100_, while in the wear area of the prosthesis used for 6 years, it increases to 380 HV_100_, which is more than 5% hardening of the material due to the cyclic load. For the femoral component, a mean increase in hardness from 400 HV_100_ for the unused prosthesis to 410 HV_100_ was observed after 6 years of use. A much more dramatic increase in hardness can be observed on the components in contact in the artificial joint over 12 years, whereas for the tibial component, the hardness in the wear area is 430 HV_100_, which accounts for a nearly 20% increase in hardness. In the case of the femoral component after 12 years of use, hardness increased from 400 HV_100_ to 440 HV_100_. The evaluation of the stress intensity coefficient demonstrated that the contact surface becomes more brittle for longer periods of use. For the unused tibial component, K_IC_ is 106 [MPa∙m^1/2^] and, with the extension of the time of use, it changes to K_IC_ = 100 [MPa∙m^1/2^] after 6 years of use, and K_IC_ = 77 [MPa∙m^1/2^] after 12 years of use. For the femoral component, the stress intensity coefficient varied from K_IC_ = 126 [MPa∙m^1/2^] for the unused component to K_IC_ = 98 [MPa∙m^1/2^] after 12 years of use. The material becomes more brittle and prone to crushing. The pattern of the use is being transformed substantially. The hard and less elastic areas of the layer have completely different characteristics as a result of tribological wear due to cyclic fatigue deformations.

The contact surfaces show numerous cracks and an increase in the width of the intergranular boundaries, which also indicates the occurrence of fatigue-type damage in this area. Pressing of M_23_C_6_ carbides into the grain boundary can be observed in the area of use. These carbides are in the entire volume of the material both at the grain boundaries and inside the grains. Furthermore, the additional Cr_23_C_6_ carbide secretions are distributed both inside the grains and at their boundaries. As a result of cyclic deformation, this hard-pressed material will not only chip and (as an artefact) be moved and pressed into another place thus increasing local wear, but will also expose the intergranular boundary.

## Figures and Tables

**Table 1:** Percentages of chemical elements.

Co [%]	Cr [%]	Mo [%]	Si [%]	C [%]
63.17	27.67	4.94	0.79	3.42

**Table 2:** Summary of the measurements of mechanical properties.

Sample Tested	HV_100_	Re [MPa]	K_IC_ [MPa•m^1/2^]
**Tibial Component, New**			
**Sample 1** **Sample 2**	365 360 359 360 362 358 Mean 360 Standard Deviation 2.50	122 120 119 120 120 119 Mean 120 Standard Deviation 1.09	108 104 106 105 108 105 Mean 106 Standard Deviation 1.67
**Tibial Component Used for 6 Years**			
**Sample 1** **Sample 2** **Sample 3**	376 378 380 384 380 378 377 388 382 Mean 380 Standard Deviation 3.81	125 126 127 128 127 126 126 129 127 Mean 127 Standard Deviation 1.20	97 101 102 100 101 102 99 97 101 Mean 100 Standard Deviation 1.94
**Tibial Component Used for 12 Years**			
**Sample 1**	432 428 431 Mean 430 Standard Deviation 2.52	144 142 14 Mean 143 Standard Deviation 1	77 77 77 Mean 77 Standard Deviation 0
**Unused Tibial Component**			
**Sample 1** **Sample 2**	397 400 394 407 406 396 Mean 400 Standard Deviation 5.40	132 133 131 136 136 131 Mean 133 Standard Deviation 2.25	130 129 122 125 127 123 Mean 126 Standard Deviation 3.22
**Femoral Component Used for 6 Years**			
**Sample 1** **Sample 2** **Sample 3**	411 406 405 411 416 415 407 411 411 Mean 410 Standard Deviation 3.77	137 135 135 137 139 138 135 137 137 Mean 137 Standard Deviation 1.41	120 128 131 124 122 121 123 118 121 Mean 123 Standard Deviation 4.07
**Femoral Component Used for 12 Years**			
**Sample 1**	437 444 438 Mean 440 Standard Deviation 3.78	145 148 146 Mean 147 Standard Deviation 1.15	100 96 98 Mean 98 Standard Deviation 2
